# HCV Specific IL-21 Producing T Cells but Not IL-17A Producing T Cells Are Associated with HCV Viral Control in HIV/HCV Coinfection

**DOI:** 10.1371/journal.pone.0154433

**Published:** 2016-04-28

**Authors:** Sonya A. MacParland, Saleh M. Fadel, Vesna Mihajlovic, Ali Fawaz, Connie Kim, A. K. M. Nur-ur Rahman, Jun Liu, Rupert Kaul, Colin Kovacs, Jason Grebely, Gregory J. Dore, David K. Wong, Mario A. Ostrowski

**Affiliations:** 1 Departments of Immunology and Medicine, University of Toronto, Toronto, Ontario, Canada; 2 Toronto General Hospital, Toronto, Ontario, Canada; 3 Maple Leaf Clinic, Toronto, Ontario, Canada; 4 The Kirby Institute, UNSW Australia, Sydney, Australia; 5 Keenan Research Centre for Biomedical Science of St. Michael’s Hospital, Toronto, Ontario, Canada; University of Montreal Hospital Research Center (CRCHUM), CANADA

## Abstract

**Background:**

Decreased hepatitis C virus (HCV) clearance, faster cirrhosis progression and higher HCV RNA levels are associated with Human Immunodeficiency virus (HIV) coinfection. The CD4^+^ T helper cytokines interleukin (IL)-21 and IL-17A are associated with virus control and inflammation, respectively, both important in HCV and HIV disease progression. Here, we examined how antigen-specific production of these cytokines during HCV mono and HIV/HCV coinfection was associated with HCV virus control.

**Methods:**

We measured HCV-specific IL-21 and IL-17A production by transwell cytokine secretion assay in PBMCs from monoinfected and coinfected individuals. Viral control was determined by plasma HCV RNA levels.

**Results:**

In acutely infected individuals, those able to establish transient/complete HCV viral control tended to have stronger HCV-specific IL-21-production than non-controllers. HCV-specific IL-21 production also correlated with HCV viral decline in acute infection. Significantly stronger HCV-specific IL-21 production was detected in HAART-treated coinfected individuals. HCV-specific IL-17A production was not associated with lower plasma HCV RNA levels in acute or chronic HCV infection and responses were stronger in HIV coinfection. HCV-specific IL-21/ IL-17A responses did not correlate with microbial translocation or fibrosis. Exogenous IL-21 treatment of HCV-specific CD8^+^ T cells from monoinfected individuals enhanced their function although CD8^+^ T cells from coinfected individuals were somewhat refractory to the effects of IL-21.

**Conclusions:**

These data show that HCV-specific IL-21 and IL-17A-producing T cells are induced in HIV/HCV coinfection. In early HIV/HCV coinfection, IL-21 may contribute to viral control, and may represent a novel tool to enhance acute HCV clearance in HIV/HCV coinfected individuals.

## Introduction

Due to shared risk factors, individuals infected with human immunodeficiency virus (HIV) are at increased risk of also being infected with hepatitis C virus (HCV) [1]. The estimated risk of cirrhosis has been found to be two-fold higher in HIV/HCV coinfected individuals compared to HCV monoinfected individuals [2]. As well, plasma HCV RNA levels are higher and the frequency of spontaneous viral clearance is lower in HIV/HCV coinfected individuals compared to HCV monoinfected persons [3–5]. It is clear from chimpanzee studies that HCV-specific T cell responses correlate with control of HCV viremia [6, 7]. In HIV/HCV coinfection, HCV-specific T cell responses are compromised, resulting in poor HCV control and more rapid liver disease progression [8].

IL-21 is a member of the common γ-chain cytokine family, produced mainly by CD4^+^ T helper cells, which regulates various innate and adaptive immune cells involved in viral clearance including T cells, B cells, NK cells, NKT and Th17 cells [9–12]. Previous work has shown that IL-21-producing T cell responses are instrumental in the control of virus infections [11, 13–15]. IL-21 has also been shown to enhance Th17 responses [9]. In the mouse LCMV infection system, which models chronic infection, CD8^+^ T cells require IL-21 to sustain effector function and resolve infection [14]. In HIV infection, the frequency of circulating HIV-specific IL-21-producing CD4^+^ T cells correlates significantly with the ability to control HIV [11, 13]. Specifically, HIV-induced secretion of IL-21 by CD4^+^ T cells leads to improved functionality and cytolytic activity of virus-specific CD8^+^ T cells [13]. Acute resolving HCV monoinfection is characterized by an expansion of IL-21 producing CD4^+^ T cells [16]. In chronic HCV monoinfection, serum IL-21 and the ability of CXCR5^+^CD4^+^ T cells to produce IL-21 are both diminished [17]. Currently, the impact of IL-21-producing T cells in HIV/HCV coinfection is unclear.

IL-17A is a member of the IL-17 family of cytokines and has been shown to induce and mediate proinflammatory effects [18]. IL-17A is secreted mainly by activated CD4^+^ T cells, including Th17 cells [10, 19]. IL-17A-producing T cell responses have been previously associated with bacterial infections and inflammatory disorders [10]. IL-17A is of interest when studying HIV/HCV coinfection as it has been shown to possess pro-fibrogenic properties, driving transforming growth factor (TGF)-β receptor upregulation on hepatic stellate cells [20]. In HIV monoinfected persons, HIV-specific, IL-17A-producing CD4^+^ T cells have been detected in early infection and have been proposed to be virus-specific T cells aberrantly primed as a result of microbial translocation due to HIV-related depletion of gut CD4 T cells [18]. HCV-specific IL-17A producing T cell responses have also been described in HCV monoinfection [21, 22] and were associated with a protective role in disease progression [21], while others have found no correlation [23]. Since Th17 cells have the ability to produce both IL-21 and IL-17A, and considering the fact that IL-21 has a known role in viral control [10], it raises the question of whether these cytokines are produced in an antigen-specific manner in HIV/HCV coinfection. The presence of HIV coinfection may negate the effect of Th17 cells on HCV viral control given that HIV also preferentially infects and eliminates Th17 cells, particularly at the mucosal surface, which may lead to the depletion HCV-specific Th17 cells in HIV untreated individuals [24].

Given the above questions regarding the IL-21/ IL-17A axis in viral control, the current study investigated the role of HCV-specific IL-17A and IL-21-producing cells in HIV/HCV coinfection in HCV viral control and liver disease progression.

## Materials and Methods

### Study Participants

We studied two cohorts: one cross-sectional and one longitudinal, which comprised individuals with acute or chronic HCV infection with or without HIV infection.

Cross-sectional cohort: Individuals were recruited from three institutions: University Health Network (Toronto, Canada), St. Michael’s Hospital (Toronto, Canada) and St Vincent’s Hospital (Sydney, Australia). Chronic HCV infection was defined as HCV sero-positivity > 6 months, associated with the presence of HCV RNA in plasma; acute HCV infection was defined as recent HCV seroconversion and detectable HCV RNA within 6 months of a negative HCV antibody test. Chronic HIV Infection was defined as HIV seropositive for > 1 year. None of the study subjects had yet received antiviral treatment for HCV. Six categories of HCV infected individuals were included in the cross-sectional study (1) Chronic HCV monoinfection, n = 10; (2) Chronic HCV/HIV coinfection, highly active anti-retroviral therapy (HAART) naïve, n = 6; (3) Chronic HCV/HIV coinfection, HAART treated, n = 7; (4) Acute HCV, chronic HIV HAART-naïve, n = 5; (5) Acute HCV, chronic HIV, on HAART n = 11; (6) acute HCV monoinfection, n = 1.

Longitudinal cohort: Seven subjects with both acute HCV infection and with chronic HIV coinfection and one subject with acute HCV monoinfection were followed longitudinally. Participants had multiple blood samplings drawn at diagnosis, and monthly or every 2 months for 6 months thereafter. HCV RNA levels were measured using Cobas Amplicor HCV Monitor v2.0 (Roche, Basel Switzerland;Conversion Factor: 1 IU/mL = 2.7 viral genome copies) or the Versant HCV RNA 3.0 (Bayer, Wuppertal Germany; Conversion factor: 1 IU/mL = 5.2 viral genome copies).

Plasma and PBMC isolated from blood taken from all individuals were cryopreserved for future use. All study participants provided informed, written consent and the research ethics boards at the University Heath Network, St. Michael’s Hospital and St Vincent’s Hospital approved the study protocol.

### Transwell cytokine secretion assay

Crypopreserved Ficoll-Hypaque isolated peripheral blood mononuclear cells (PBMC) were thawed and CD8^+^ T cell depleted to avoid absorption of cytokines from supernatants by CD8^+^ T cells in the assay with Dynal CD8^+^ depletion kits (Life Technologies, Carlsbad, CA) resulting in >90% depletion of CD8^+^ T cells in PBMC samples (not shown). 0.5 x 10^6^ cells were then plated in duplicate in the bottom chamber of a 96-well HTS transwell plate (3μm pore size) (Corning Inc., Corning, NY) in 100μL of RPMI 1640 medium (Life Technologies) supplemented with 10% FCS, L-glutamine and penicillin/streptomycin (Life Technologies). Duplicate wells were stimulated for 6 days with either 1μg/mL Staphylococcal Enterotoxin B (SEB) (Toxin Technologies, Sarasota, FL); 1μg/mL HCV NS3 antigen (Mikrogen GmbH, Munich, Germany); 1μg/mL HCV NS4 antigen (Mikrogen), 1μg/mL HCV Core antigen (Austral Biologicals, San Ramon, CA) or medium alone. Anti-CD28 (BD Pharmingen, Franklin Lakes, New Jersey) and anti-CD49d (Biolegend, San Diego, CA), (both at 1μg/ml) were added with the antigens for costimulation. Anti-IL-21 and IL-17A antibody coated polycarbonate beads were added to the upper chamber after 72 hours (Millipore Corporation, Billerica, MA). At day 6 of culture, beads in the upper chambers were washed twice and analyzed on a Bioplex Reader (Bio-Rad laboratories, Redmond, WA).

### In vitro culture with recombinant IL-17A and IL-21

Cryopreserved PBMC were resuspended in RPMI 1640 media (Life Technologies) supplemented with 10% fetal bovine serum, 1% L-glutamine and 1% penicillin-streptomycin at 2 x 10^6^ cells/ml in a 48-well round-bottom plate. HLA-A2^+^ subjects were identified using HLA-A2 antibody staining (BD Pharmingen) and subsequent flow cytometry analysis. PBMC from each sample were cultured in medium alone or medium supplemented with recombinant human (rh) IL-21 (at 100 ng/ml) (ebiosciences, San Diego, CA), or rh-IL-17A (at 100 ng/ml)(Miltenyi Biotech, Bergisch Gladbach, Germany) for 120 hours at 37°C and 5% CO_2_.

After 120 hours, cells were transferred to a 96-well V-bottom plate and stimulated separately with either medium plus DMSO alone (negative control) or 1μg/pep/mL of 10 overlapping 18-mers spanning HCV NS3 1073 (National Institute of Health Reagent Program, Bethesda, MD) at 1μg/mL per peptide for 6 hours at 37°C and 5% CO_2_ in the presence of monensin and brefeldin A (1:1000) (BD Biosciences). Anti-human CD28 (BD Pharmingen) and anti-human CD49d (Biolegend) (both at 1 μg/ml) antibodies were added for costimulation. As a positive control, cells were separately stimulated with anti-CD3 (Biolegend) and anti-CD28 (Biolegend) (both at 1 μg/ml).

### Multicolor cytokine flow cytometry

Crypopreserved PBMC were stained with fluorophore-conjugated monoclonal antibodies to human CD4 (Biolegend OKT-4), CD8 (Biolegend 53–6.7), IL-21R (BD Biosciences 17A12) or an IgG1 isotype control (ebioscience P3). Cells were washed and fixed with BD Cyotofix/Cytoperm fixation buffer (BD Biosciences). Cells were then washed with Perm/Wash buffer (BD Biosciences) and intracellular cytokine staining was performed using anti-IFN-γ (Biolegend B27). An aqua amine dye (Invitrogen) was used as a discriminating marker for live and dead cells. Background for intracellular cytokine staining was calculated based on Fluorescence-minus-one (FMO) and no-peptide negative controls. Data were acquired on an LSRII flow cytometer using BD FACSDiva software (both BD Biosciences) and analyzed by FlowJo (Tree Star Inc., San Carlos, CA).

### LPS and soluble CD14 assays

Lipopolysaccharide (LPS) assays (limulus amebocyte lysate assay kit, Lonza, Bazel, Switzerland) were performed according to the manufacturer’s instructions. Briefly, plasma samples were diluted 5 times in endotoxin-free water, heat inactivated at 70°C for 10 minutes, and assayed to quantify plasma LPS levels. A commercially available enzyme-linked immunosorbent assay (ELISA) kit was used to measure plasma levels of soluble CD14 (Quantikine Human soluble CD14 Immunoassay; R&D Systems, Minneapolis, MN).

### Statistical Analysis

All data were analyzed using Graphpad Prism version 5.0 software (GraphPad Software, San Diego, CA). Differences in median IL-21 levels, IL-17A levels, LPS levels, and soluble CD14 levels were measured using the two-tailed Mann-Whitney U test. The impact of recombinant IL-21 treatment on rescue of HCV-specific T cell responses was evaluated using the two-tailed paired T-test for paired samples. Correlations were described using the Spearman’s rank correlation coefficient. For subject characteristics, means and standard deviations for each subject group were calculated and analyzed by one-way ANOVA with Bonferroni’s multiple comparison post-test to determine whether there were significant differences between groups.

## Results

### Participant Characteristics

We studied 8 individuals longitudinally during acute HCV infection and the other patients were sampled at a single time point during chronic HCV infection. Since up to 25% of individuals with acute HCV infection can eventually control virus, longitudinal study of these individuals could provide insights into immune correlates of virus control. 7 HIV/HCV coinfected individuals and 1 HCV monoinfected individual, all infected with HCV genotype 1 or 1a, were prospectively followed. 2 of the 8 patients exhibited spontaneous HCV viral control and 1 exhibited partial viral control and were included in the “partial and complete controllers” group, defined as having plasma HCV RNA levels of <1,000 IU/ml at one or more time points during acute infection. These 3 individuals had the following HCV RNA levels during acute infection: OM525- presented with a viral RNA level of 129IU/ml and 4 and 5 months after presentation had undetectable HCV RNA levels; OM634-presented with a viral RNA level of 42 IU/ml, and had HCV RNA levels of 1.3 x 10^4^ IU/mL 3 months after presentation and 3.4 x 10^4^ at 5 months after presentation, at which point this individual elected to initiate antiviral therapy (pegylated IFN-α2a/Ribavirin); OM755-presented with a viral RNA level of 1.42x 10^6^ IU/mL and became HCV RNA undetectable when tested 4 and 5 months after presentation. The 5 individuals that progressed to chronic infection, the non-controller group, were defined as having plasma HCV RNA levels of >1,000 IU/ml at all time points during acute infection. The cross-sectional study included a total of 39 subjects with chronic HCV monoinfection, chronic HCV infection with HIV coinfection, and acute HCV infection with HIV coinfection (Table 1). Individuals were stratified by HCV infection state (acute versus chronic) and HAART treatment status. All individuals were HCV therapy-naïve at the time of the study. Baseline and nadir CD4^+^ T cell counts, and duration of HAART were similar between groups. ALT levels were higher in acute HCV infection. HAART treated HIV/HCV coinfected individuals had the highest fibrotest scores however these subject were also older. HIV coinfected subjects tended to have higher plasma HCV RNA levels.

**Table 1:** Participant Characteristics.

	HCV mono (n = 10)	Chronic HIV/HCV Co (N = 13)		Acute HIV/HCV Co (N = 16)		
		On HAART (n = 6)	Not on HAART (n = 7)	On HAART (n = 11)	Not on HAART (n = 5)	Sig.
**Age (y, Mean (St. Dev))**	40.78 (11.44)	43.60 (12.10)	29.00 (11.05)	46.44 (8.52)	31.40 (7.67)	**0.02**
**Sex (no. male)**	9	6	6	7	5	
**HCV genotype (no. type 1)**	9	6	7	10	3	
**HCV plasma VL (IU**^#^**/ml, Mean (St. Dev))**	5.69E+06 (8.24E+06)	8.70E+06 (1.05E+07)	2.44E+07 (3.48E+07)	5.59E+05 (9.13E+05)	5.90E+06 (9.56E+06)	**n.s.**
**HIV plasma VL (copies/ml, Mean (St. Dev))**	na	576.83 (1190.94)	34184.57 (35273.71)	<50	38208.33 (29417.35)	**0.012**
**CD4 T cell Count (Cells/mm3, Mean (St. Dev))**	na	702.00 (371.57)	654.57 (290.51)	578.75 (257.10)	492.20 (305.07)	**n.s.**
**ALT U/ml, Mean (St. Dev))**	50.70 (19.20)	71.00 (31.54)	67.50 (72.41)	145.33 (132.54)	397.75 (441.79)	**0.027**
**Fibrotest score, Mean (St. Dev))**	0.36 (0.25)	0.59 (0.32)	0.18 (0.08)	n.t	nt	**0.047**
**Duration of HCV infection (Years, Mean (St. Dev))**	13.39 (11.56)	7.67 (6.50)	3.63 (2.70)	<0.5	<0.5	**n.s.**
**Duration of HIV infection (Years, Mean (St. Dev))**	n/a	17.67 (7.03)	4.17 (2.23)	8.70 (4.87)	3.10 (3.05)	**0.0002**
**Nadir CD4 T cell Count (Cells/mm**^**3**^**, Mean (St. Dev))**	n/a	281.50 (175.68)	570.17 (249.83)	343.40 (120.30)	506.25 (337.03)	**n.s.**
**Duration of HAART (Years, Mean (St. Dev))**	n/a	9.17 (3.97)	n/a	7.00 (6.68)	n/a	n.s.

HCV mono: HCV monoinfected; HIV/HCV Co: HIV/HCV Coinfected; VL: Viral Load; ALT: Alanine transaminase; HAART: Highly active antiretroviral therapy; nt: Not tested. Means and standard deviations for each patient group were calculated and analyzed by one-way ANOVA with a Bonferroni multiple comparison post test to determine whether there were significant differences between groups.

^#^1 IU = 2.7 HCV viral genome copies.

### HCV-specific IL-21-producing responses in acute infection are associated with relative viral control

**Fig 1:**
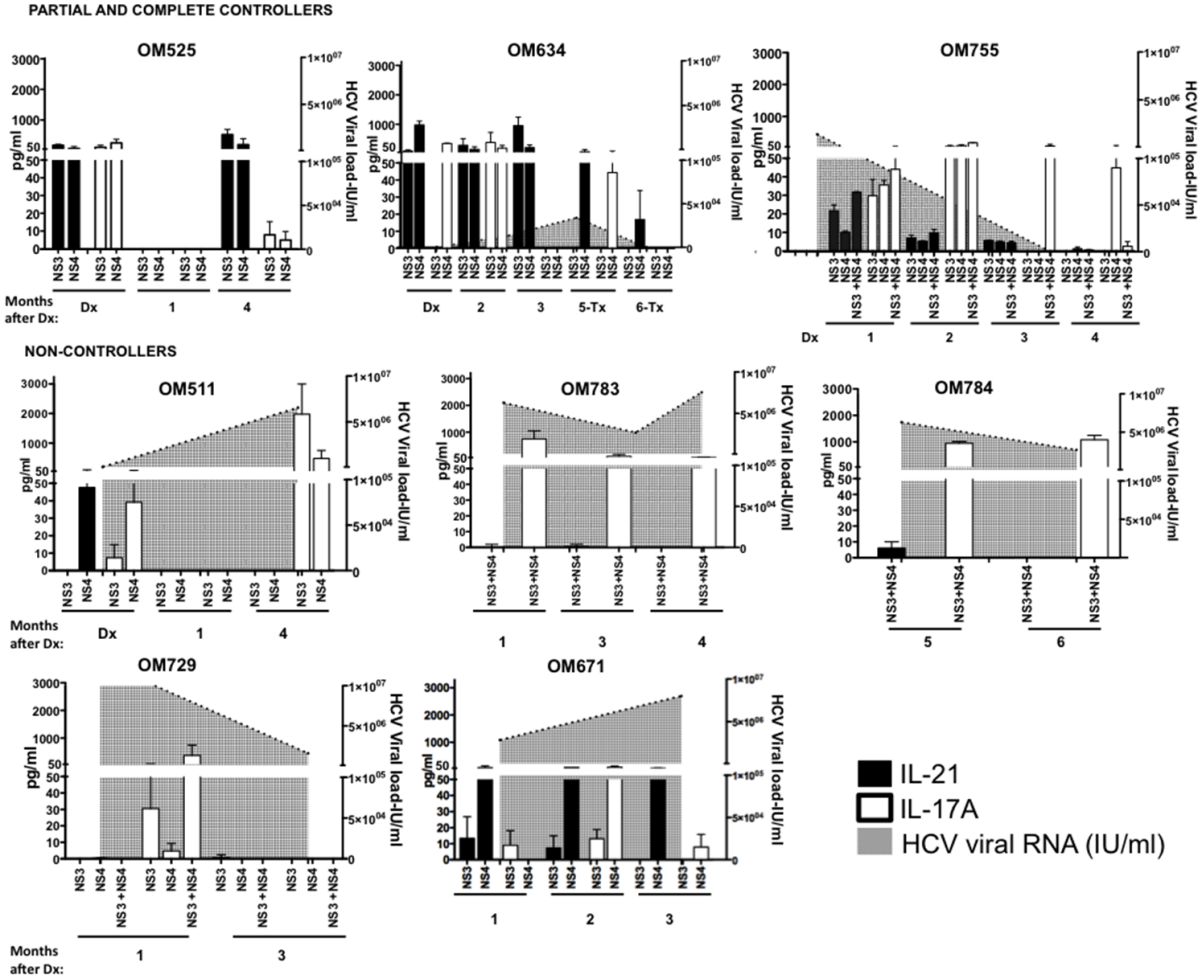
HCV-specific IL-21 secretion is associated with relative HCV control in acute infection. HCV-specific IL-21 and IL-17A secretion was examined in acutely HCV-infected individuals who controlled or transiently controlled (Partial and Complete Controllers) or did not control (Non-Controllers) HCV infection. Partial and Complete Controllers included two acutely HCV infected patients with ongoing HAART-treated HIV infection (OM525, OM634) and one individual who was acutely HCV monoinfected (OM755). Non-controllers included four acutely HCV infected patients with HAART-treated HIV infection (OM511, OM729, OM783, OM784) and one individual with acute HCV infection and untreated HIV infection (OM671). HCV-specific responses from acutely HCV-infected individuals who controlled or did not control HCV were examined longitudinally. Black bars show HCV-specific IL-21 secretion, white bars show HCV-specific IL-17A secretion. Left y-axis shows cytokine secretion (represented by bars), right y-axis shows HCV RNA levels (represented by grey shaded area). Assays carried out in duplicate showing standard error.

In order to determine the role of HCV-specific IL-21 and IL-17A producing CD4^+^ T cells in HCV control we performed a highly sensitive transwell microbead-based cytokine capture assay to measure IL-21 and IL-17A production in antigen-stimulated cells as previously described [13]. In this transwell assay, CD8^+^ T cells are depleted from total PBMC as done previously [13] to remove the bias of enrichment of CD8^+^ T cells in PBMC from HIV infected individuals and to increase the sensitivity of IL-21 detection with the removal of IL-21 receptor expressing CD8^+^ T cells. As illustrated in Fig 1, we prospectively followed 7 HIV/HCV coinfected individuals and 1 HCV monoinfected individual, all infected with HCV genotype 1 or 1a. Of these 8 acute HCV infected individuals, 3 exhibited either spontaneous clearance or transient viral control (controllers) while the remaining individuals progressed to chronic infection. One of the individuals in the controller group (OM634) was treated with antiviral therapy (pegylated IFN-α2a/Ribavirin) for HCV after reaching a plasma HCV RNA level of 3.4 x 10^4^ IU/mL. One of the non-controllers (OM671) had a history of recent acquisition of HIV infection for 8 months prior to being diagnosed with HCV infection. In all three acute HCV individuals who exhibited transient (OM634) or complete (OM525, OM755) viral control, IL-21 secretion was induced to both HCV NS3 and NS4 proteins at presentation and subsequent intervals in follow-up (See Fig 1). In the 5 acutely HCV infected individuals unable to control viremia (Fig 1), there were few IL-21 responses detected and in one individual only (OM671) was there IL-21 producing responses against multiple HCV antigens at multiple time points. In contrast, IL-17A was produced after HCV antigen stimulation in both those that controlled and those that did not control infection, at presentation and subsequent time points.

### Individuals with HIV/HCV coinfection on HAART therapy exhibit stronger peripheral HCV-specific IL-21 responses

**Fig 2:**
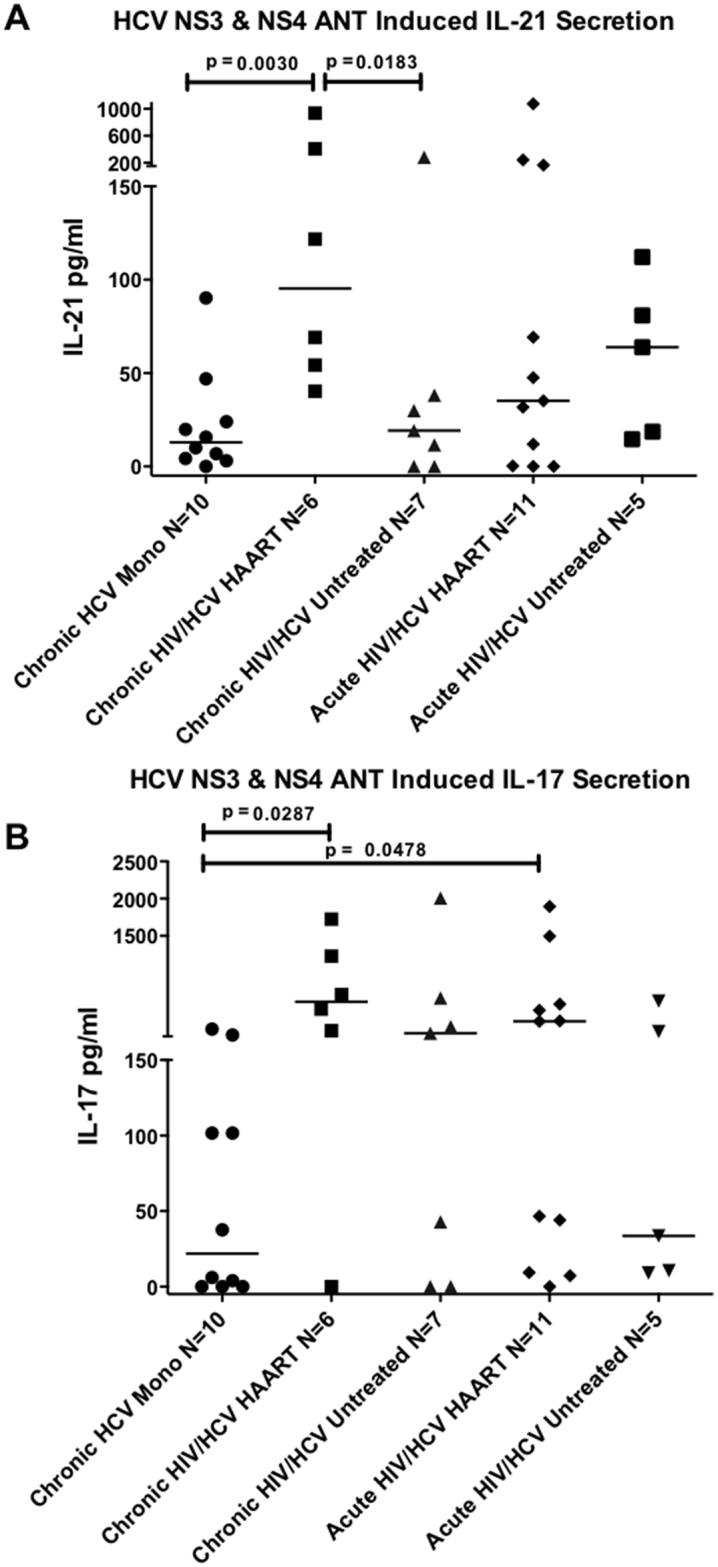
HIV/HCV coinfected individuals on HAART therapy exhibit stronger peripheral HCV-specific IL-17A and IL-21 responses. HCV-specific IL-21 and IL-17A secretion was examined in HCV monoinfected (Chronic HCV Mono) and HIV/HCV coinfected persons. Coinfected subjects included 6 individuals with chronic HCV and HAART-treated HIV infection (Chronic HIV/HCV HAART), 7 individuals with chronic HCV and HAART-naïve HIV infection (Chronic HIV/HCV Untreated), 11 individuals with acute HCV and HAART-treated HIV infection (Acute HIV/HCV HAART), and 5 individuals with acute HCV and HAART-naïve HIV infection (Acute HIV/HCV Untreated). HCV specific IL-21 secretion (**A**) and anti-IL-17A secretion (**B**) was examined. Samples were analyzed in duplicate, values represent combined HCV NS3 and HCV NS4 -induced IL-21 or IL-17A secretion minus background in DMSO only wells. Median values are indicated in the figure. Statistical significance was calculated using the two-tailed Mann-Whitney U test. P< 0.05 considered statistically significant.

We then compared HCV specific IL-21 and IL-17A responses in individuals with acute versus chronic HCV infection, with or without HIV infection, and on or off HAART, using the above described transwell microbead-based cytokine capture assay. In the cross-sectional study, we examined HCV-induced secretion of IL-21 and IL-17A from the PBMC of individuals with HCV monoinfection, and acute and chronic HCV infection on a background of chronic HIV infection using the above described transwell microbead-based cytokine capture assay. In Fig 2, we saw that HCV-specific IL-21 production was significantly higher in the PBMC of chronically HCV/HCV coinfected individuals on HAART therapy compared to HCV monoinfected individuals and HIV/HCV coinfected individuals not on HAART (*p* = 0.003; *p* = 0.0183 respectively, Fig 2A). Stronger HCV-specific IL-21 producing responses were also observed in the PBMC of acute HCV infected individuals with pre-existing HAART-treated HIV infection although it did not reach significance. In terms of HCV-specific IL-17A production, we observed no significant differences in IL-17A production at the different stages of HIV/HCV coinfection, however there was an overall trend to more HCV-specific IL-17A producing responses in the PBMC of individuals with HIV/HCV coinfection compared to those with HCV monoinfection with HCV-specific IL-17A production being significantly higher in the PBMC of individuals with chronic and acute HCV with ongoing HAART-treated HIV infection compared to those with HCV monoinfection (*p* = 0.0287; *p* = 0.0478, respectively) (Fig 2B).

### HCV-specific IL-21-producing responses in acute infection are associated with viral control

**Fig 3:**
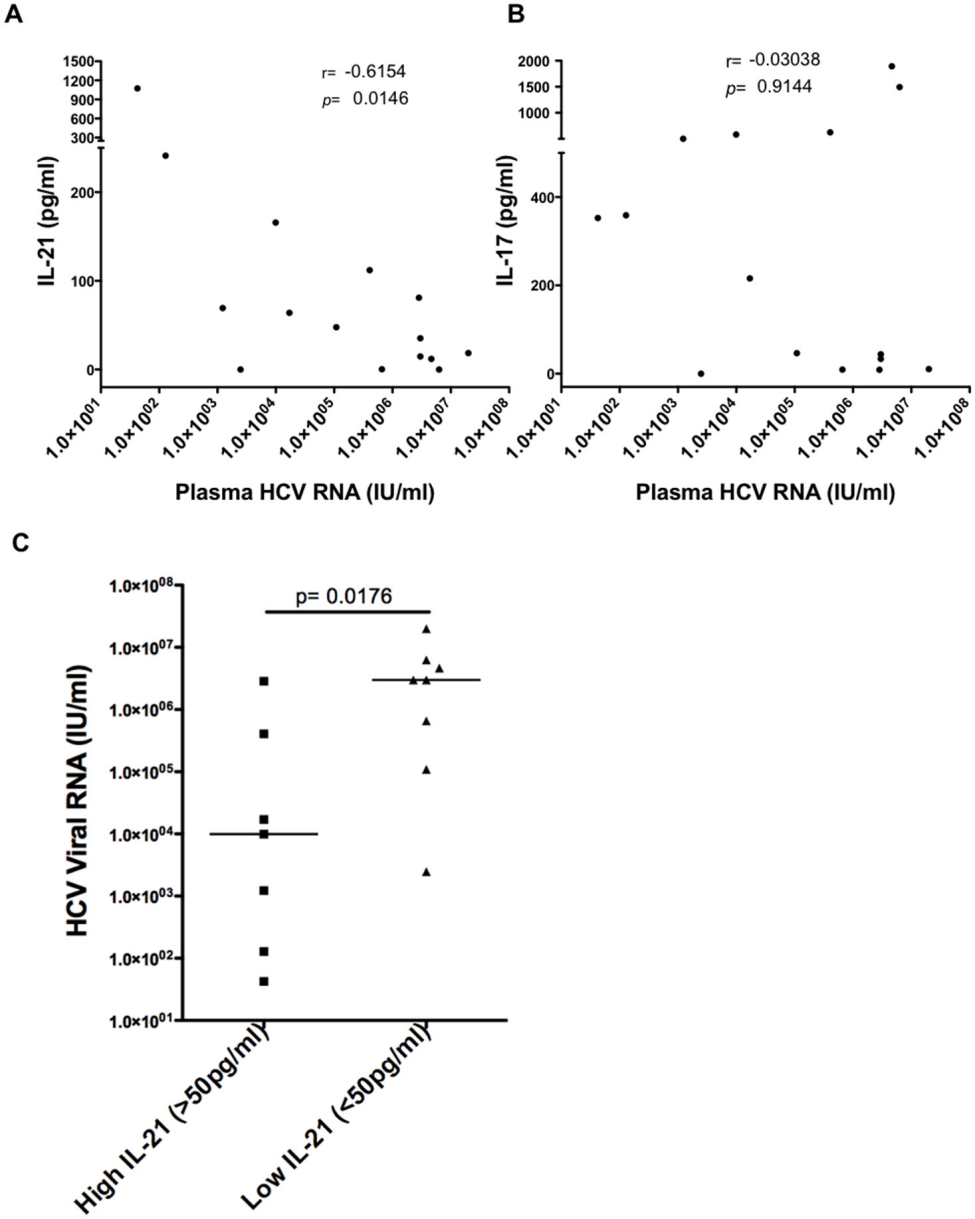
HCV-specific IL-21 responses correlate with HCV RNA levels in acute HIV/HCV coinfection. Correlations between combined HCV NS3 and HCV NS4-induced IL-21 (**A**) and IL-17A (**B**) secretion from the PBMC of coinfected subjects and RNA levels were evaluated in individuals with acute HCV infection with prior HIV infection at the time of presentation. Statistical analyses were carried out using the Spearman’s rank correlation test. (C) Acutely HCV-infected patients were stratified as having low HCV-specific IL-21 production (<50pg/ml) and high HCV-specific IL-21 production (>50pg/ml) and differences in HCV RNA levels were examined. Median values are indicated in the figure. Statistical significance was calculated using the two-tailed Mann-Whitney U test. *p*< 0.05 considered statistically significant.

We compared HCV-specific IL-17A and IL-21 production with plasma HCV RNA levels in individuals with acute HCV infection with prior HIV infection (Fig 3). HCV-specific IL-21 producing responses were inversely correlated with plasma HCV RNA levels (r = -0.615, *p* = 0.0146) (Fig 3A), however no correlation was seen between HCV-specific IL-17A responses and plasma HCV RNA levels (Fig 3B). We then stratified the acutely infected patients based on the amount of IL-21 being secreted from PBMC in response to HCV antigens. As seen in Fig 3C, plasma HCV RNA levels in individuals with HCV-specific IL-21 producing responses >50pg/ml were significantly lower than plasma HCV RNA levels in individuals with HCV-specific IL-21 producing responses <50pg/ml. We then examined the relationship between HCV-specific IL-17A and IL-21 production and plasma HCV RNA levels in individuals sampled during chronic HCV mono- and chronic HIV/HCV coinfection and saw no correlation between HCV-specific IL-21 or IL-17A responses and plasma HCV RNA levels (S1A–S1D Fig) suggesting that in chronic infection, there may be a defect in the ability of IL-21 to activate HCV-specific CD8^+^ T cells. We were also unable to observe a correlation between HCV-specific IL-21 producing responses and the magnitude of *ex vivo* HCV-specific CD8^+^ T cell responses in individuals during chronic HCV mono- and chronic HIV/HCV coinfection although we saw a trend towards higher HCV-specific IFN-gamma production from CD8^+^ T cells of acutely infected patients with stronger IL-21 responses in CD4^+^ T cells (S1E–S1G Fig).

### LPS Levels are elevated in the plasma of individuals with HIV/HCV coinfection on HAART therapy

**Fig 4:**
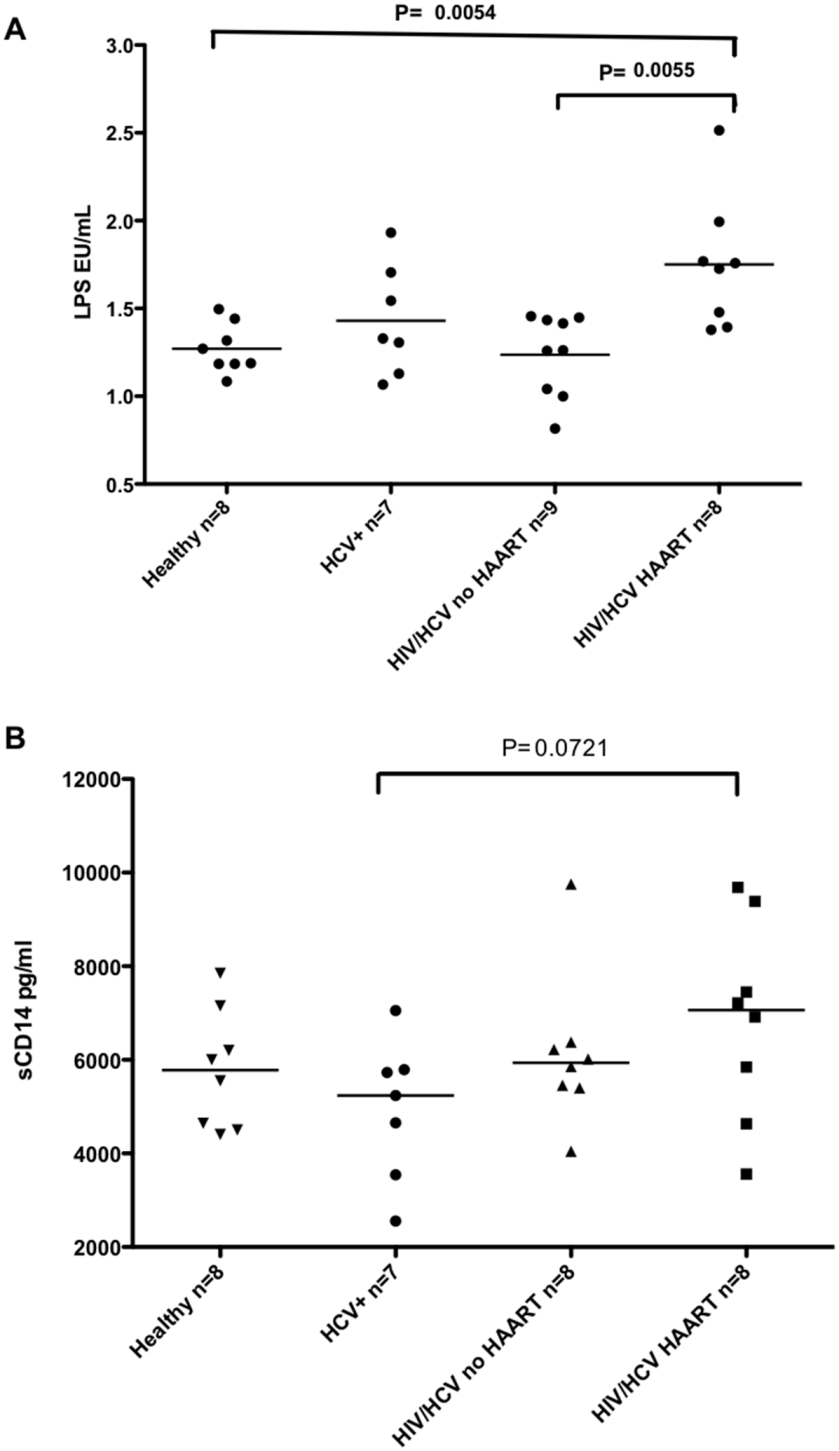
Serum LPS levels are higher in HAART-treated individuals and may stimulate IL-21 responses. **A)** LPS Levels measured in heat-inactivated plasma and B) Soluble CD14 levels measured in plasma of HIV/HCV coinfected individuals on HAART therapy (HIV/HCV HAART), treatment naïve (HIV/HCV no HAART), HCV monoinfected (HCV Mono), compared to HIV/HCV naïve individuals (HEALTHY). Assays were performed in duplicate. Median values are indicated in the figure. Statistical significance was calculated using the two-tailed Mann-Whitney U test. P< 0.05 considered statistically significant.

Considering previous work suggesting that Th17 cells may be induced due to gut microbial translocation, we sought to examine LPS and soluble CD14 levels in the plasma of our cross-sectional cohort using the limulus amebocyte lysate assay and sCD14 ELISA. Fig 4A illustrates that HIV/HCV coinfected individuals on HAART treatment had significantly higher levels of plasma LPS when compared to healthy individuals and to HIV/HCV coinfected individuals not on HAART (p = 0.0054 and p = 0.0055, respectively). We also measured plasma soluble CD14 levels as an additional marker of microbial translocation and found a trend towards higher sCD14 in plasma of HAART-treated coinfected individuals compared to their HCV-monoinfected counterparts (Fig 4B).

### HCV-specific IL-21 and IL-17A responses are found in the liver during HCV infection

Although, we could detect HCV-specific IL-17A and IL-21-producing T cells in the peripheral blood in most individuals, it is unclear whether these cells can be targeted to the liver where virus is replicating. In an individual with chronic HCV monoinfection (genotype 1a, HCV RNA level 1.08 x 10^6^ IU/mL and ALT 248 U/L) we examined contemporaneous *ex vivo* PBMC and intrahepatic lymphocytes (S2 Fig). In this individual, we found HCV-specific IFN-γ and IL-17A, IL-21 production from intrahepatic lymphocytes but only IFN-γ and IL-17A produced by PBMC in response to HCV antigens (S2 Fig). In both peripheral blood and hepatic tissues, HCV specific responses producing IFN-γ and IL-17A outweighed those producing IL-21. The liver biopsy showed Metavir stage 3 fibrosis in this individual. Thus, IL-21 and IL-17A can be produced in the liver in response to HCV antigens although IL-21 production was much lower in this individual with chronic HCV infection.

### HCV-specific IL-21 and IL-17A responses do not correlate with Fibrotest score or microbial translocation

We compared peripheral HCV-specific IL-21 and IL-17A-producing T cell responses with a well-established marker of liver progression, the Fibrotest, as well as LPS levels, a marker of microbial translocation in those with chronic HCV/HIV coinfection. There were no significant correlations between HCV specific IL-21 and IL-17A-producing T cell responses and either LPS levels or Fibrotest scores (See S3 & S4 Figs).

### *In vitro* recombinant IL-21 treatment leads to the rescue of *ex vivo* HCV-specific IFN-γ producing CD8^+^ T cell responses

**Fig 5:**
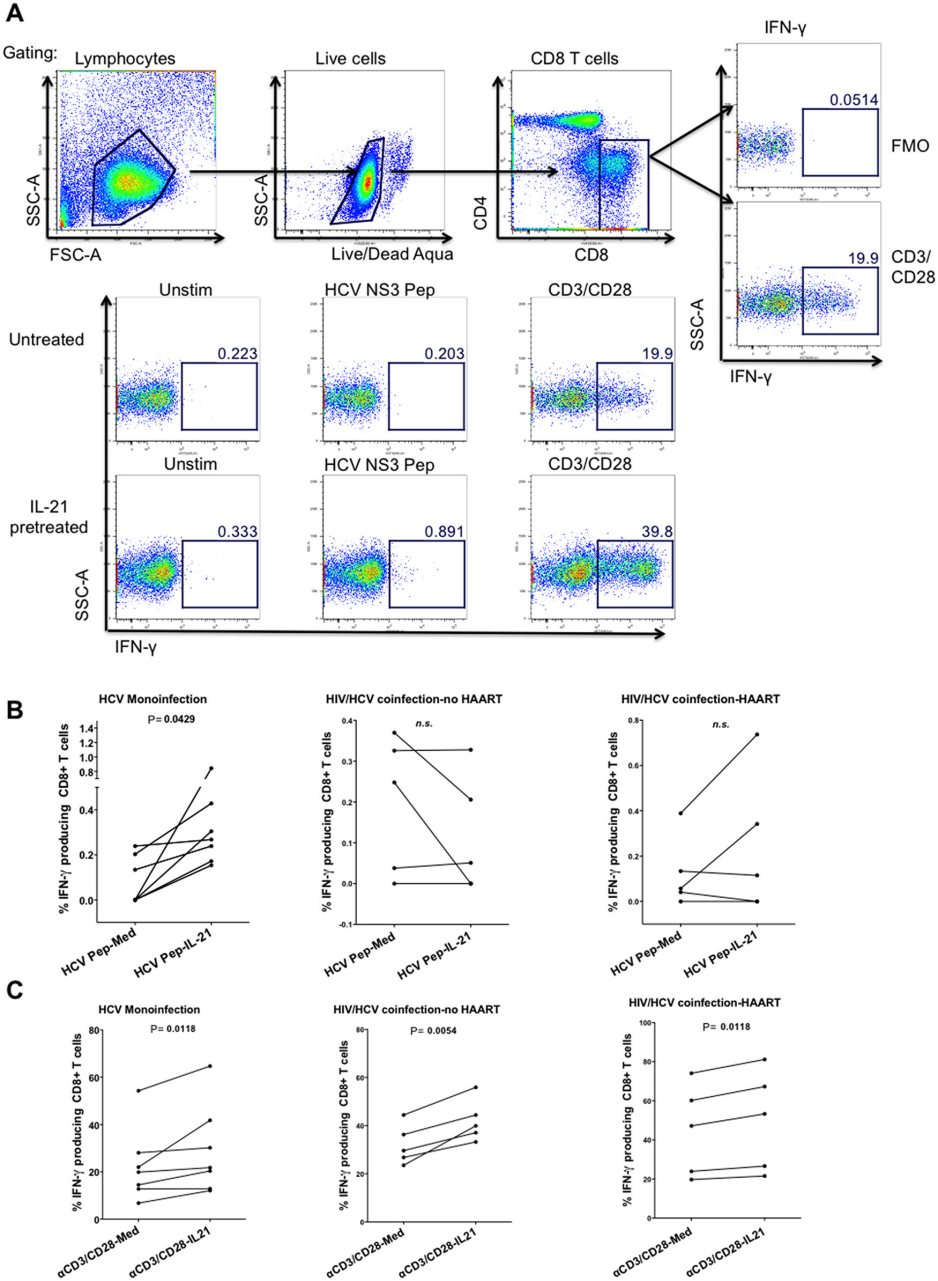
Recombinant IL-21 treatment leads to the rescue of HCV-specific CD8^+^ T cell responses. PBMCs from 7 chronically HCV monoinfected individuals and 10 HIV/HCV coinfected individuals were cultured in media alone or with recombinant human IL-21 (at 100 ng/ml) for 120h. PBMC were then pulsed with media alone plus DMSO (Unstim) or 1μg/pep/mL of 10 overlapping 18-mers spanning HCV NS3 1073 (HCV NS3 Pep), or anti-CD3/anti-CD28 (CD3/CD28). Intracellular IFN-γ was measured by intracellular cytokine staining. A) Representative flow cytometry plots of HCV-specific IFN-γ produced by T cells cultured in media alone or with recombinant human IL-21. Summary of flow cytometry data showing IFN-γ secreted by B) HCV peptide pulsed and C) CD3/CD28 stimulated CD8^+^ T cells. IFN-γ induction displayed as percentage of IFN-γ production from CD8^+^ T cells minus no peptide background (Unstim). Statistical significance was calculated using 2-tailed paired T-test. P< 0.05 considered statistically significant.

**Fig 6:**
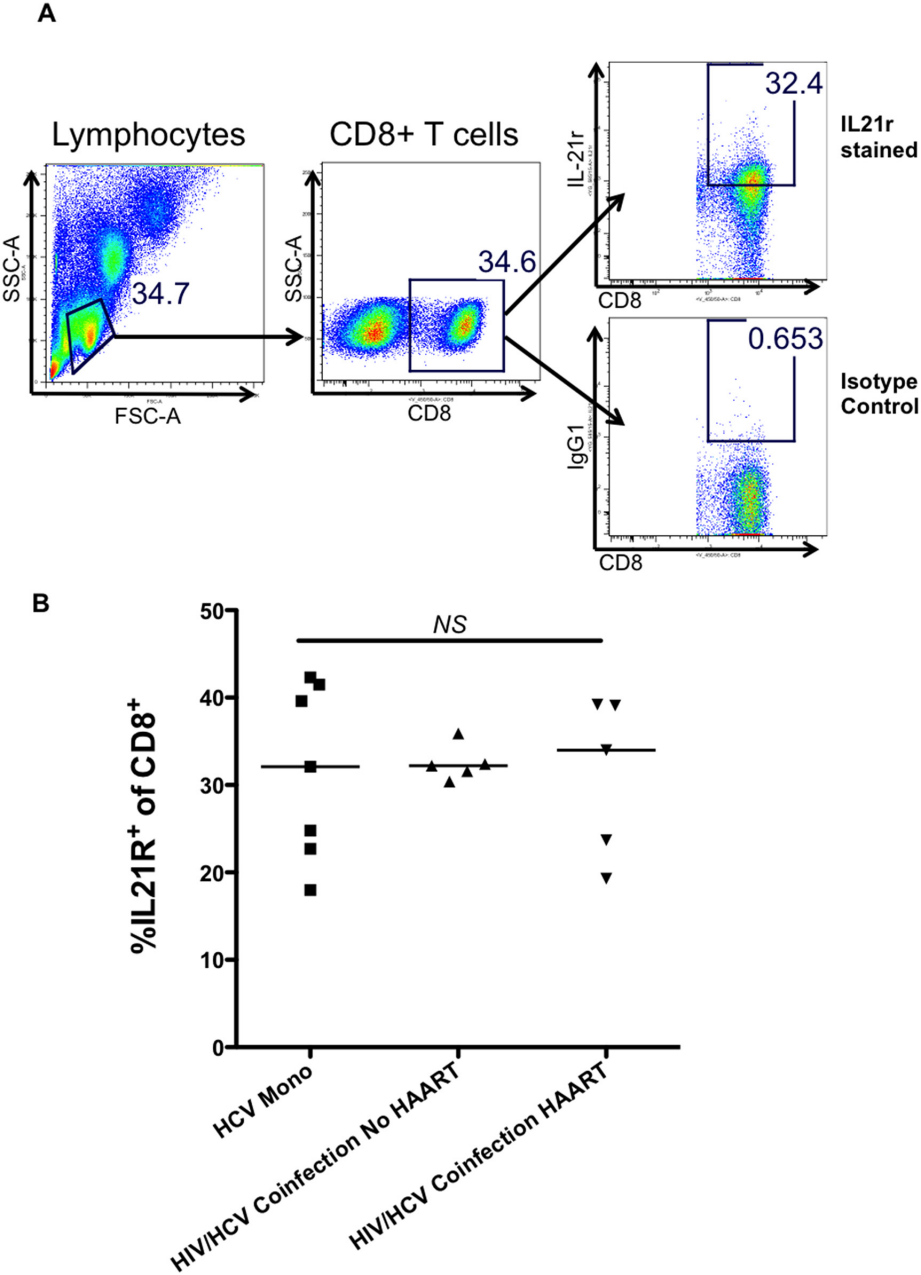
No significant difference between IL21R expression in CD8+ T cells from HIV/HCV coinfected individuals compared to HCV monoinfected individuals. IL-21R expression was examined by flow cytometry in CD8+ T cells from the PBMC of HIV/HCV coinfected subjects in the chronic phase of HCV infection who were receiving HAART (HIV/HCV coinfection HAART) or who were HAART-naïve (HIV/HCV Coinfection No HAART) compared to individuals with chronic HCV monoinfection (HCV Mono). A). Representative flow cytometry plots showing detection of IL-21R expression in CD8+ T cells. B) Summary of flow cytometry data. Median values are indicated in the figure. Statistical significance was calculated using the two-tailed Mann-Whitney U test. P< 0.05 considered statistically significant.

To explore the ability of IL-21 to enhance the function of HCV-specific CD8^+^ T cells, we cultured PBMC in the presence of recombinant IL-17A and IL-21 prior to the stimulation of HCV-specific CD8^+^ T cells with HCV peptides. The HCV-specific response was measured by percentage of IFN-γ producing CD8^+^ T cells in response to HCV peptides over background. For every HCV monoinfected individual, IL-21 treatment of PBMC enhanced production of IFN-γ from HCV-specific CD8^+^ T cells (p = 0.0429) (See Fig 5A and 5B). In the group with HIV/HCV coinfection, we saw little to no improvement in HCV-specific CD8^+^ T cell IFN-γ responses in those who were HAART naïve, and enhancement of responses in 2/5 HAART-treated individuals (Fig 6). Pretreatment with recombinant IL-21 enhanced CD3/CD28 stimulated IFN-γ production from CD8^+^ T cells in all three groups of patients, suggesting that the refractoriness observed in the PBMC of coinfected individuals is restricted to the HCV-specific CD8^+^ T cells (See Fig 5C). Recombinant IL-17A treatment did not enhance HCV-specific IFN-γ production from CD8^+^ T cells in any of the individuals studied (data not shown). A different response to IL-21 could be due to IL-21 receptor alpha (R) expression differences, however we did not observe differences in IL-21R expression on CD8^+^ T cells between HCV monoinfected and HIV/HCV coinfected individuals (See Fig 6). Thus, IL-21 can improve HCV-specific CD8^+^ T cell function in PBMC from individuals with HCV monoinfection. PBMC from individuals with HIV/HCV coinfection are somewhat refractory to IL-21 treatment.

## Discussion

In this study, we examined HCV-specific IL-17A and IL-21 responses in HIV/HCV coinfection. These responses are of interest in HIV/HCV coinfection due to the role of virus-specific IL-21-production in the enhancement of viral clearance [11, 13–15] and the pro-fibrogenic properties of IL-17A [20]. We found that HCV-specific IL-21 production was enhanced in the PBMC of patients who exhibited relative viral control in acute HIV/HCV coinfection. In addition, HCV-specific IL-17 producing responses were stronger in the PBMC of HIV/HCV coinfected individuals compared to their monoinfected counterparts, although these responses remained strong regardless of the ability of the individual to control viremia. The fact that HCV-specific IL-21 responses correlated with HCV viral control in acute HIV/HCV coinfection is consistent with previous findings from HIV, HBV and HCV monoinfections in which virus-specific IL-21 production was associated with viral control [11, 13, 15, 25] and suggests that HCV-specific IL-21 secretion may rescue HCV-specific CD8^+^ T cell responses, leading to viral decline.

In chronic HIV/HCV coinfection, HCV-specific IL-21 responses were higher in the PBMC of patients receiving HAART therapy. Meanwhile, while not significant, the mean plasma HCV RNA levels in HAART-treated individuals were approximately 2.8 times lower than their HAART naïve counterparts, suggesting a role of IL-21 in limiting viral proliferation in not only acute but also chronic HCV infection. However, in our study, we found that stronger HCV-specific IL-21 responses correlated with better viral control in acute but not chronic infection, suggesting that HCV-specific CD8^+^ T cells may be less able to respond to IL-21 in chronic infection. HAART treatment has been associated with reduced plasma HCV RNA levels [26, 27] although not in all cases [28]. Based on our results, it is possible that HAART treatment may lead to immune reconstitution and the recovery of HCV-specific IL-21-producing T cells, which can provide help to HCV-specific CD8^+^ T cells, leading to viral decline. This suggests that early HAART treatment may benefit patients by enhancing IL-21 responses. Future studies examining responses before and after HAART would confirm this.

Although recombinant IL-21 was able to significantly rescue HCV-specific CD8^+^ T cell responses in chronically HCV monoinfected individuals, we did not see a consistent effect of IL-21 in improving HCV-specific CD8^+^ T cell responses in HIV/HCV coinfected individuals, despite similar levels of IL-21R. However, IL-21 pretreatment was able enhance CD8^+^ T cell IFN-γ secretion in response to CD3/CD28 stimulation in HIV/HCV coinfected individuals. This suggests that the presence of HIV coinfection might induce an IL-21 refractory state in the HCV-specific CD8^+^ T cells of some individuals, as a feature of T cell exhaustion [8]. Future studies will examine downstream signaling events after IL-21R binding to determine if a defect exists in this pathway that prevents CD8^+^ T cells of coinfected patients from responding to the binding of IL-21 to its receptor. Taken together, our data suggests that HCV-specific IL-21-producing responses may be favorable not only in acute infection but also in chronic HCV infection.

The fact that we did not see an association of HCV-specific IL-17A responses with relative immune control in acute infection suggests that IL-17A may be part of a cytokine milieu that favors HCV disease progression and inflammation rather than viral clearance. Indeed, it has been recently shown that IL-17A has pro-fibrogenic properties, driving hepatic stellate cell activation [20]. We also found that HAART treatment and HIV viral control did not impact the degree of IL-17A being secreted in response to HCV antigens. The impact of HAART on fibrosis progression is currently unclear, however recent data has shown that HAART-treated HCV coinfected persons have higher rates of fibrosis progression and hepatic decompensation than their HCV monoinfected counterparts [29]. In agreement with this, fibrotest scores tended to be higher in HAART-treated individuals from our cohort. It is known that Th17 cells are enriched in the intestine [30–32] and are depleted during HIV infection when there is a rapid and irreversible CD4^+^ T cell reduction in the gastrointestinal tract, resulting in the translocation of bacterial components into the gut [33]. We found that HAART-treated coinfected persons had significantly higher levels of plasma LPS when compared to healthy individuals and to HAART-naive coinfected individuals and may be due to differences in age and duration of HIV infection. HCV infection alone did not lead to higher LPS levels, a finding that confirms earlier data [34]. The finding that the differences in LPS levels in HIV/HCV coinfected treatment-naïve individuals remained insignificant in comparison to the healthy donors was surprising and may be due to a relatively younger age, shorter duration of HIV infection and higher nadir CD4^+^ T cell counts in these individuals.

In this study, we were limited by a lack of available liver tissue and thus our results are predominantly describing HCV-specific responses in the periphery, which may be distinct from that of the liver. Also, using the transwell cytokine secretion assay, we were unable to show whether the lack of HCV-specific responses was associated with the expression of exhaustion markers, as has been shown previously [35]. While we attempted to use flow cytometric techniques to examine HCV-specific CD4^+^ and CD8^+^ T cell responses, the flow-based assays were not sensitive enough to detect HCV-specific IL-21 being secreted by CD4+ T cells. This is likely due to the progressive T cell exhaustion observed in chronic HCV infection that is worsened in HIV/HCV coinfection [8, 20]. Our patient population was not age-matched which may also be a potential confounder. As well, longitudinal blood samples were acquired during follow-up appointments for acute infection and therefore in some cases only two samples were acquired. Our longitudinal studies were limited by small numbers of patients as it is challenging to capture patients during acute HCV infection. In particular, we were unable to recruit more than 1 acutely HCV monoinfected individual into our longitudinal study. As well, we were unable to include additional coinfected patients not under anti-retroviral therapy as the majority of HIV/HCV coinfected patients in our cohort were on HAART therapy or were immediately put on HAART therapy by their physician when acute HCV coinfection was detected. In conclusion, the findings from this work suggest that in HIV/HCV coinfection, IL-21 appears to act as an effector cytokine, contributing to the maintenance of HCV viral control, and may have potential as a therapy to augment or enhance refractory CD8^+^ T cell responses to improve viral control.

## Supporting Information

S1 Fig HCV-specific IL-21 responses do not correlate with HCV RNA levels or HCV-specific CD8^+^ T cell responses in chronic HCV monoinfection and chronic HIV/HCV coinfection. Correlations between plasma HCV RNA levels and combined HCV NS3 and HCV NS4-induced secretion of IL-21 (**A&C**) and IL-17A (**B&D**) from the PBMC of chronically HCV monoinfected subjects and chronically HIV/HCV coinfected subjects were evaluated. Statistical analyses were carried out using the Spearman’s rank correlation test. (**E-G**) Correlations between combined HCV NS3 and HCV NS4-induced secretion of IL-21 and HCV-specific CD8^+^ T cell responses in **E**) Chronically HCV monoinfected individuals, **F**) Acutely HCV infected individuals with prior chronic HIV Infection, **G)** Chronically HIV/HCV coinfected individuals were evaluated. IFN-γ induction displayed as percentage of IFN-γ producing CD8^+^ T cells after stimulation with 1μg/pep/mL of 10 overlapping 18-mers spanning HCV NS3 1073 minus no peptide background. Gating strategy for IFN-γ positive events shown in Fig 5A. Statistical analyses were carried out using the Spearman’s rank correlation test. P< 0.05 considered statistically significant.
(TIFF)

S2 Fig HCV-specific IL-21 and IL-17A responses are found in the liver during HCV infection. PBMC and intrahepatic lymphocytes from an HCV genotype 1 monoinfected individual with grade 3 fibrosis on liver biopsy were stimulated with antigens and assessed for HCV-specific cytokine production using an IL-17A, IL-21 and IFN-γ transwell multiplex-based secretion assay following stimulation with HCV NS3, HCV NS4 and HCV core antigens (all 1 μg/ml) or 1μg/pep/mL of 10 overlapping 18-mers spanning HCV NS3 1073 (HCV NS3 Pep). Assays were performed in duplicate showing standard errors. Values represent IL-21, IL-17A and IFN-γ secretion minus background in DMSO treated wells.
(TIFF)

S3 Fig No significant correlation between HCV specific IL-21 and IL-17A-producing T cell responses and LPS levels. A correlation between HCV-specific IL21 and IL-17A secretion from the PBMC of HIV/HCV coinfected subjects in the acute and chronic phase of HCV infection and LPS levels was evaluated in individuals with available clinical information. Statistical analyses were carried out using the Spearman’s rank correlation test. P< 0.05 considered statistically significant.
(TIFF)

S4 Fig No significant correlation between HCV specific IL-21 and IL-17A-producing T cell responses and Fibrotest score. A correlation between HCV-specific IL21 and IL-17A secretion from the PBMC of HIV/HCV coinfected subjects in the chronic phase of HCV infection and Fibrotest scores was evaluated in individuals with available clinical information. Statistical analyses were carried out using the Spearman’s rank correlation test. P< 0.05 considered statistically significant.
(TIFF)
