# Supplementary figures and images for: HCV Specific IL-21 Producing T Cells but Not IL-17A Producing T Cells Are Associated with HCV Viral Control in HIV/HCV Coinfection
Source: PLoS One. 2016 Apr 28;11(4):e0154433. doi: 10.1371/journal.pone.0154433 (PMC4849786; doi:10.1371/journal.pone.0154433)

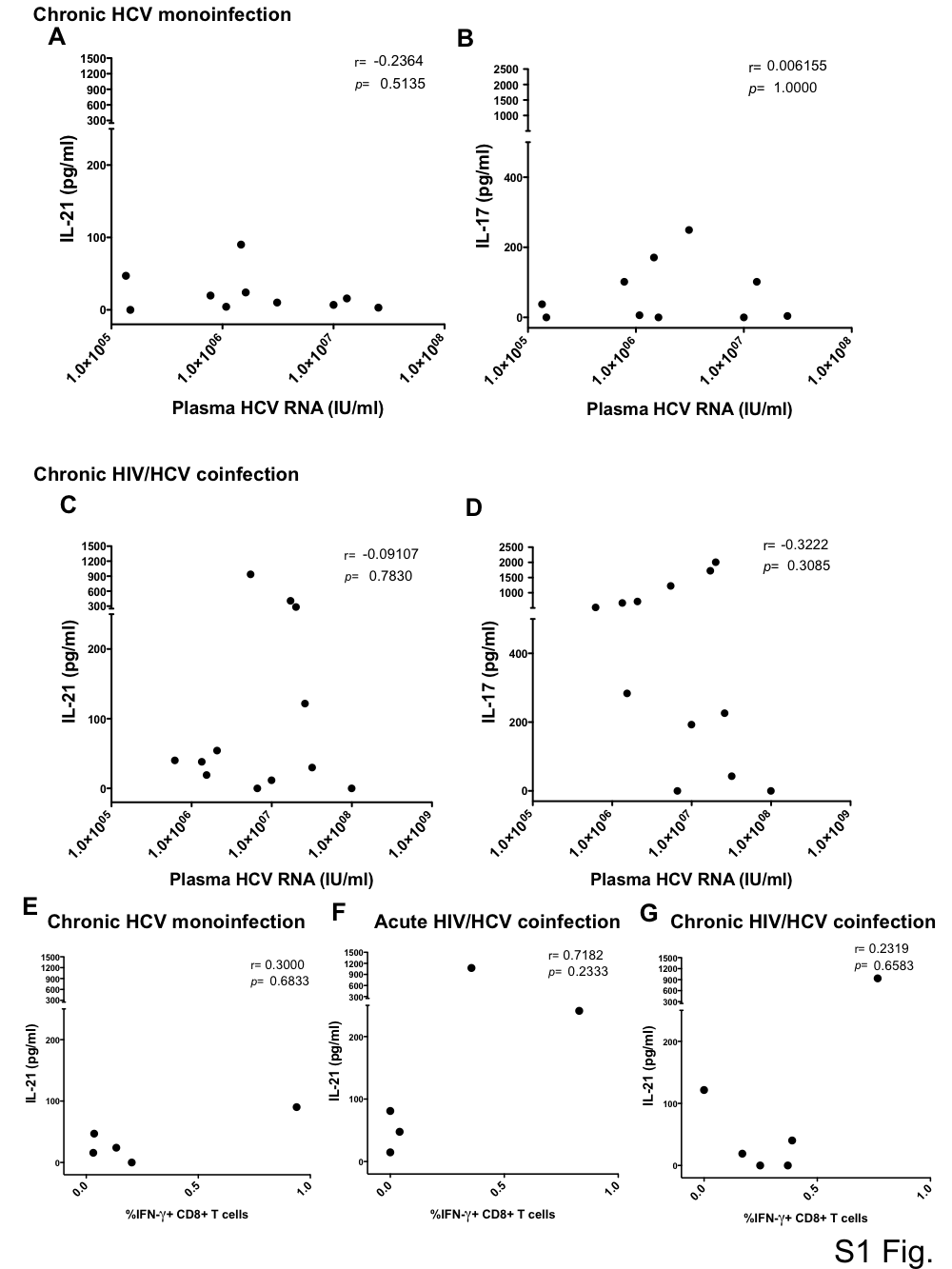

Supplement: S1 Fig — Correlations between plasma HCV RNA levels and combined HCV NS3 and HCV NS4-induced secretion of IL-21 (A&C) and IL-17A (B&D) from the PBMC of chronically HCV monoinfected subjects and chronically HIV/HCV coinfected subjects were evaluated. Statistical analyses were carried out using the Spearman’s rank correlation test. (E-G) Correlations between combined HCV NS3 and HCV NS4-induced secretion of IL-21 and HCV-specific CD8+ T cell responses in E) Chronically HCV monoinfected individuals, F) Acutely HCV infected individuals with prior chronic HIV Infection, G) Chronically HIV/HCV coinfected individuals were evaluated. IFN-γ induction displayed as percentage of IFN-γ producing CD8+ T cells after stimulation with 1μg/pep/mL of 10 overlapping 18-mers spanning HCV NS3 1073 minus no peptide background. Gating strategy for IFN-γ positive events shown in Fig 5A. Statistical analyses were carried out using the Spearman’s rank correlation test. P< 0.05 considered statistically significant. (TIFF) [file pone.0154433.s001.tiff]

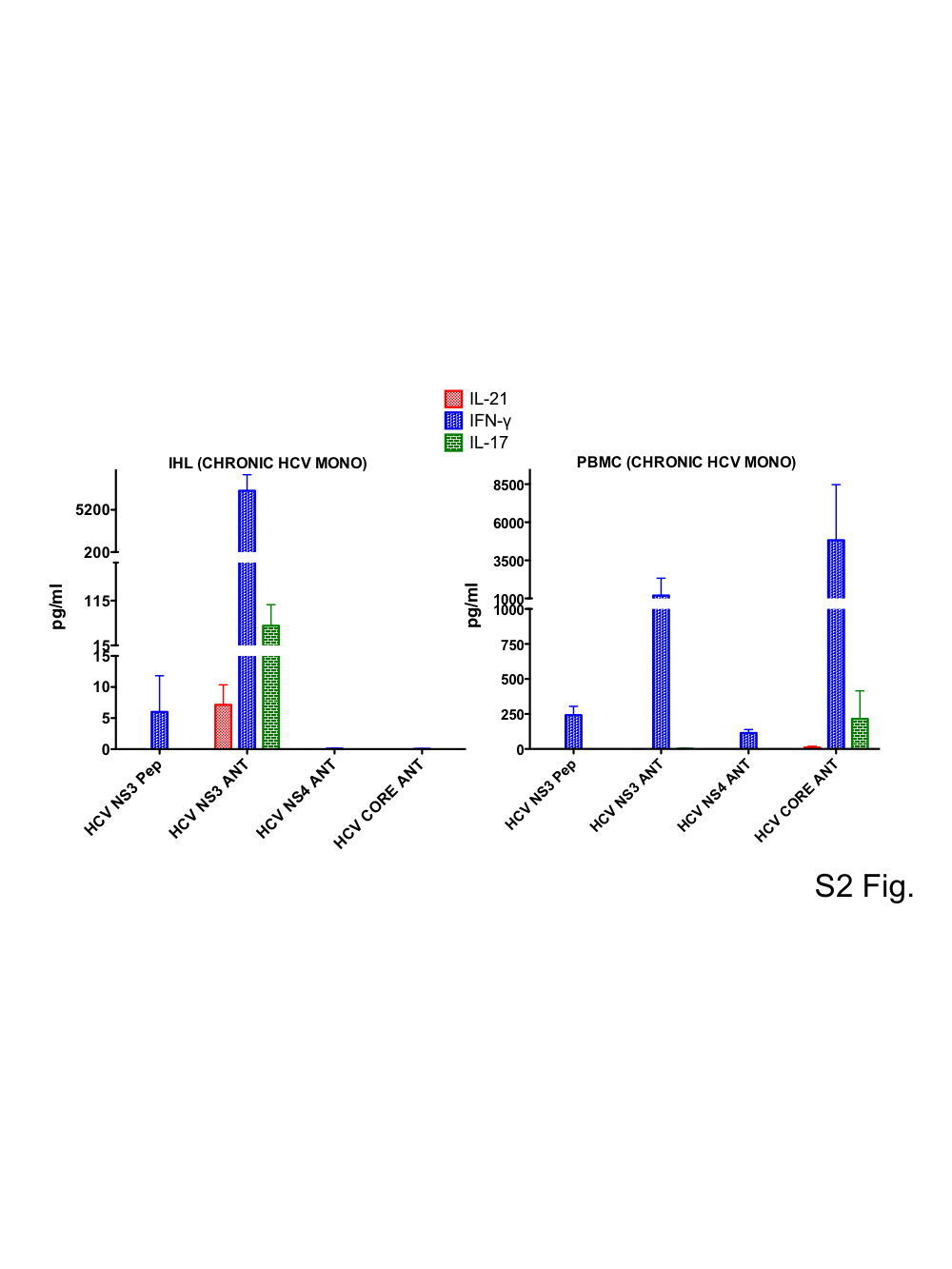

Supplement: S2 Fig — PBMC and intrahepatic lymphocytes from an HCV genotype 1 monoinfected individual with grade 3 fibrosis on liver biopsy were stimulated with antigens and assessed for HCV-specific cytokine production using an IL-17A, IL-21 and IFN-γ transwell multiplex-based secretion assay following stimulation with HCV NS3, HCV NS4 and HCV core antigens (all 1 μg/ml) or 1μg/pep/mL of 10 overlapping 18-mers spanning HCV NS3 1073 (HCV NS3 Pep). Assays were performed in duplicate showing standard errors. Values represent IL-21, IL-17A and IFN-γ secretion minus background in DMSO treated wells. (TIFF) [file pone.0154433.s002.tiff]

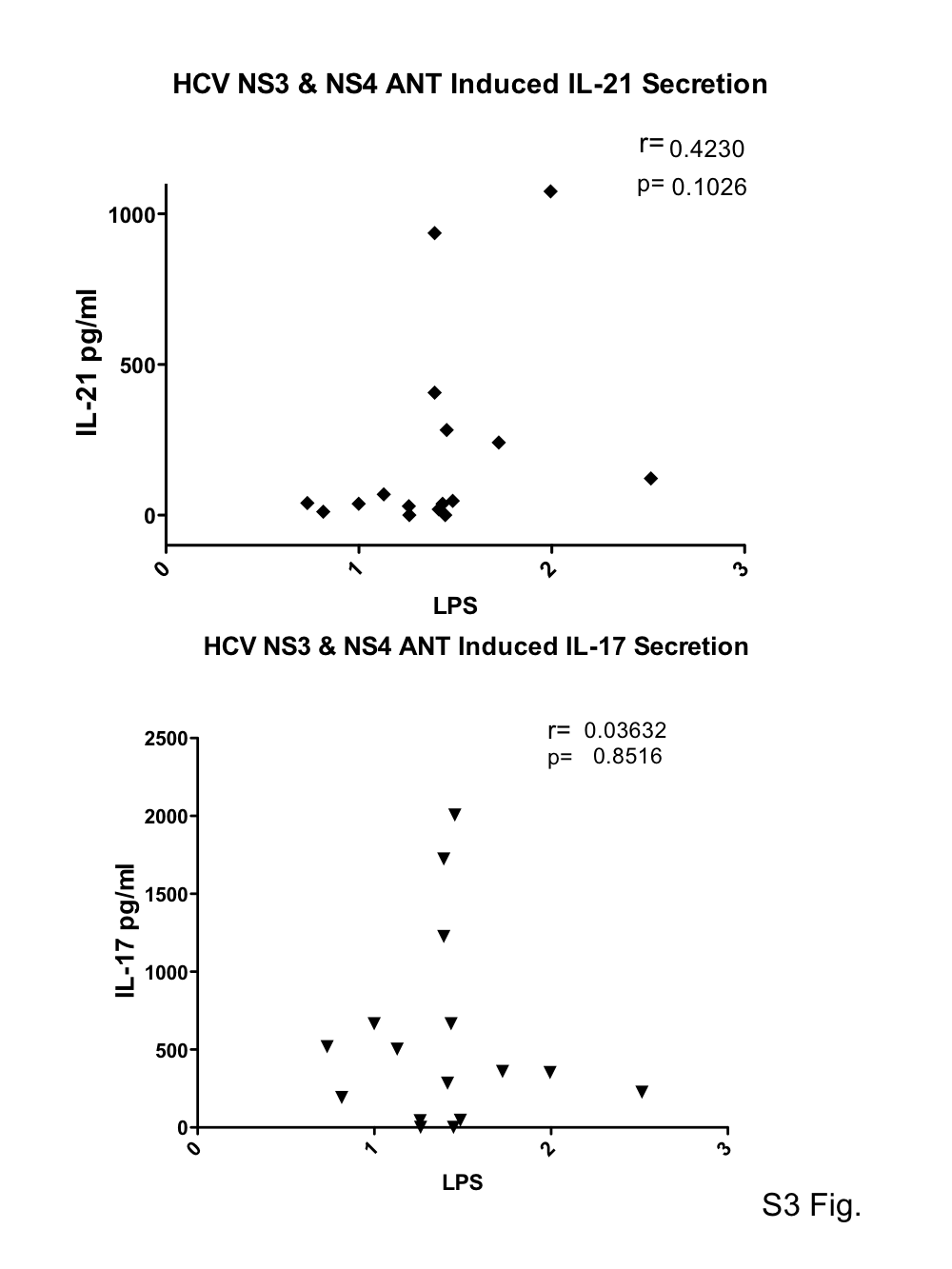

Supplement: S3 Fig — A correlation between HCV-specific IL21 and IL-17A secretion from the PBMC of HIV/HCV coinfected subjects in the acute and chronic phase of HCV infection and LPS levels was evaluated in individuals with available clinical information. Statistical analyses were carried out using the Spearman’s rank correlation test. P< 0.05 considered statistically significant. (TIFF) [file pone.0154433.s003.tiff]

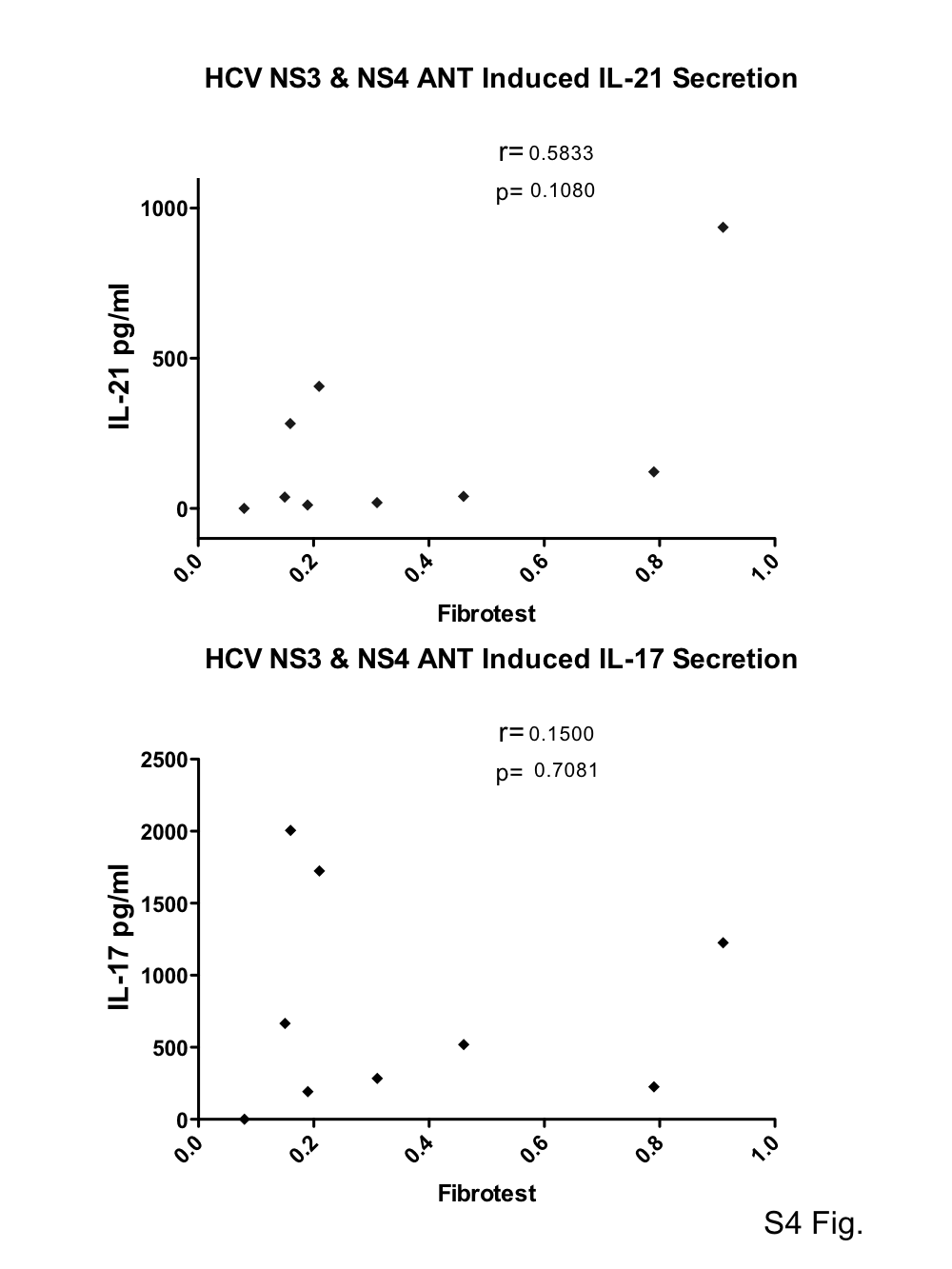

Supplement: S4 Fig — A correlation between HCV-specific IL21 and IL-17A secretion from the PBMC of HIV/HCV coinfected subjects in the chronic phase of HCV infection and Fibrotest scores was evaluated in individuals with available clinical information. Statistical analyses were carried out using the Spearman’s rank correlation test. P< 0.05 considered statistically significant. (TIFF) [file pone.0154433.s004.tiff]
